# Tumor microenvironment and cancer metastasis: molecular mechanisms and therapeutic implications

**DOI:** 10.3389/fphar.2024.1442888

**Published:** 2024-11-12

**Authors:** Cigir Biray Avci, Bakiye Goker Bagca, Masoud Nikanfar, Leila Sabour Takanlou, Maryam Sabour Takanlou, Alireza Nourazarian

**Affiliations:** ^1^ Department of Medical Biology, Faculty of Medicine, Ege University, Izmir, Türkiye; ^2^ Department of Medical Biology, Faculty of Medicine, Adnan Menderes University, Aydin, Türkiye; ^3^ Department of Neurology, Faculty of Medicine, Tabriz University of Medical Sciences, Tabriz, Iran; ^4^ Department of Basic Medical Sciences, Khoy University of Medical Sciences, Khoy, Iran; ^5^ Student Research Committee, Khoy University of Medical Sciences, Khoy, Iran

**Keywords:** tumor microenvironment, molecular mechanisms, cancer metastasis, cellular microenvironment, signal transduction

## Abstract

The tumor microenvironment (TME) plays a crucial role in cancer development and metastasis. This review summarizes the current research on how the TME promotes metastasis through molecular pathways, focusing on key components, such as cancer-associated fibroblasts, immune cells, endothelial cells, cytokines, and the extracellular matrix. Significant findings have highlighted that alterations in cellular communication within the TME enable tumor cells to evade immune surveillance, survive, and invade other tissues. This review highlights the roles of TGF-β and VEGF signaling in promoting angiogenesis and extracellular matrix remodeling, which facilitate metastasis. Additionally, we explored how metabolic reprogramming of tumor and stromal cells, influenced by nutrient availability in the TME, drives cancer progression. This study also evaluated the therapeutic strategies targeting these interactions to disrupt metastasis. By providing a multidisciplinary perspective, this study suggests that understanding the molecular basis of the TME can lead to more effective cancer therapies and identify potential avenues for future research. Future research on the TME should prioritize unraveling the molecular and cellular interactions within this complex environment, which could lead to novel therapeutic strategies and personalized cancer treatments. Moreover, advancements in technologies such as single-cell analysis, spatial transcriptomics, and epigenetic profiling offer promising avenues for identifying new therapeutic targets and improving the efficacy of immunotherapies, particularly in the context of metastasis.

## 1 Introduction

Metastasis, the spread of cancer cells from the primary tumor to distant organs, remains a significant challenge in cancer treatment. A critical factor in this process is the tumor microenvironment (TME), which is a complex system composed of various cellular and non-cellular components (Nallasamy et al., 2022). The TME includes stromal cells, the extracellular matrix (ECM), immune cells, and soluble factors, all interacting to support cancer cell survival, proliferation, and dissemination (Tiwari et al., 2022; Shen and Kang, 2018). Metastasis is a multistep process involving invasion, intravasation, circulation, survival in the bloodstream, extravasation, and secondary tumor formation. Interactions between cancer cells and TME regulate each step (Majidpoor and Mortezaee, 2021). Understanding the molecular mechanisms of the TME and its influence on metastasis is crucial for developing effective therapeutic strategies. The TME is not a passive backdrop for tumor growth; it actively shapes cancer progression through numerous mechanisms. For example, stromal cells such as fibroblasts can differentiate into cancer-associated fibroblasts (CAFs) (Baghban et al., 2020). These CAFs remodel the ECM, promote angiogenesis, and secrete growth factors and cytokines that enhance tumor cell invasion and metastasis (Neophytou et al., 2021). The ECM itself undergoes significant alterations in the TME, becoming more conducive to cancer cell movement and providing biochemical signals that drive metastatic behavior (Brassart-Pasco et al., 2020). Immune cells within the TME, including macrophages, T cells, and neutrophils, can either suppress or promote tumor growth and metastasis, depending on their state of activation and the signals they receive from the tumor and its surroundings (Ramos et al., 2022). Metastasis involves a multistep process, including local invasion, intravasation into the bloodstream, survival in circulation, extravasation into distant tissues, and the establishment of secondary tumors. Each of these steps is regulated by the interactions between cancer cells and the TME (Link et al., 2021). For instance, during local invasion, cancer cells must degrade and navigate through the ECM facilitated by matrix metalloproteinases (MMPs) secreted by both cancer and stromal cells (Gui and Bivona, 2022). Cancer cells evade immune surveillance in the bloodstream, which is often aided by platelets, which cloak them and provide survival signals. Upon reaching distant organs, cancer cells encounter a new microenvironment that can either support or hinder their growth into secondary tumors (Baghban et al., 2020).

**FIGURE 1:**
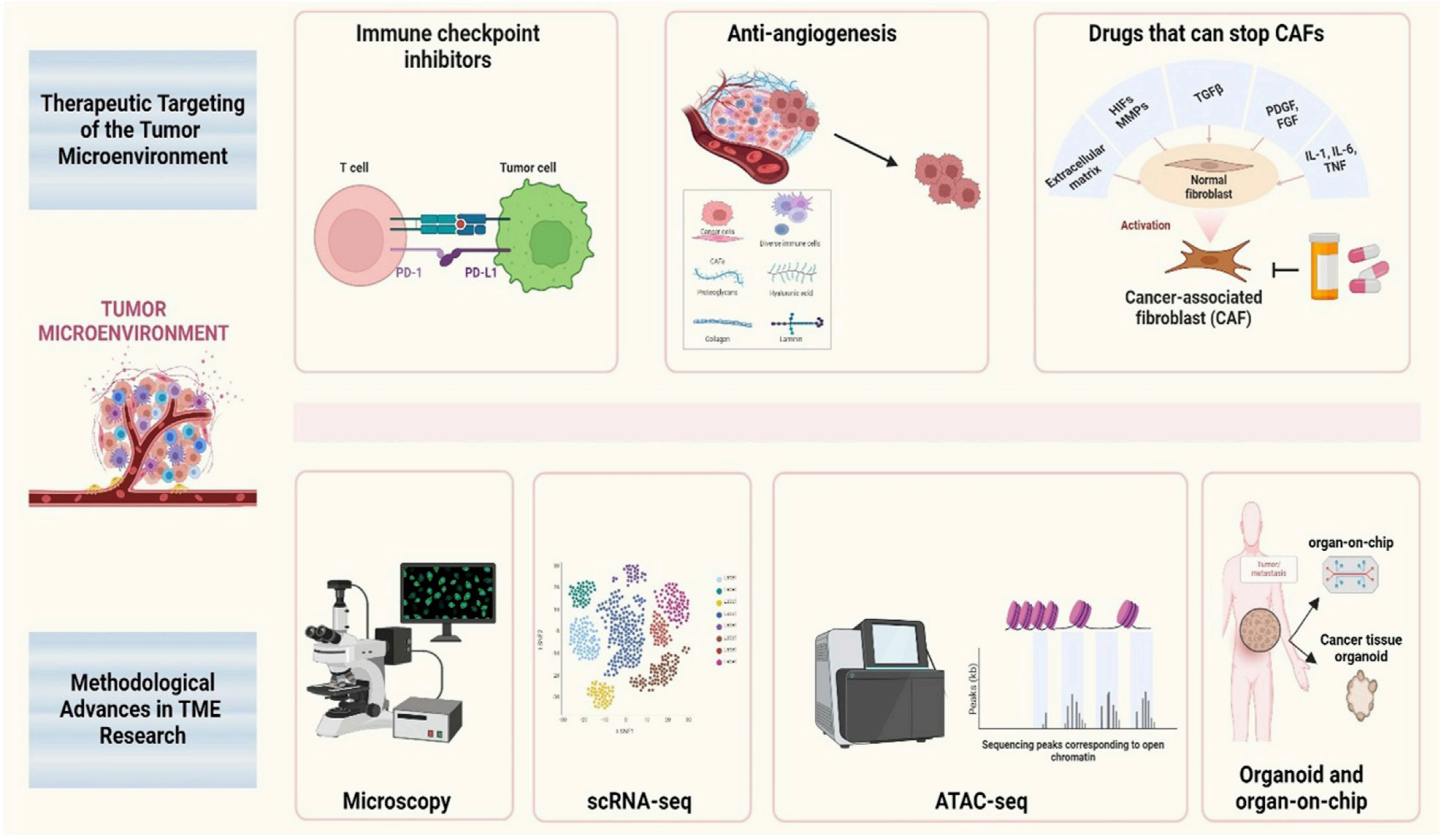
Schematic therapeutic targeting and methodological advances in TME research This figure illustrates various therapeutic strategies and methodological advances in the study of the TME, with a particular focus on the key areas of intervention and research tools used to analyze its dynamics. The schematic highlights the integration of therapeutic approaches targeting cancer-associated fibroblasts, immune cells, and angiogenesis, as well as the application of advanced methodologies such as single-cell RNA sequencing, spatial omics, and live imaging techniques.

Studying the role of TME in cancer metastasis is crucial for multiple reasons. First, TME significantly influences cancer cell behavior, including invasion, survival in the bloodstream, and colonization of distant organs. Understanding these interactions may reveal new targets for therapeutic interventions. Second, targeting the TME offers a potential strategy for disrupting the metastatic process and improving patient outcomes (Neophytou et al., 2021). Recent research has highlighted the importance of molecular pathways and signaling networks within the TME that regulate metastasis. Pathways involving growth factors (*e.g.,* TGF-β, VEGF, and EGF), cytokines (*e.g.,* IL-6 and TNF-α), and chemokines (*e.g.,* CXCL12) play critical roles in modulating the behavior of both cancer and stromal cells (Zalpoor et al., 2022). Additionally, hypoxic conditions commonly found within tumors can activate hypoxia-inducible factors (HIFs) that drive angiogenesis and metabolic adaptations favoring metastasis (Bae et al., 2024). Advancements in the methodology and therapeutic targeting in TME research are shown in Figure 1.

This review aims to address several gaps in the current understanding of the role of TME in cancer metastasis. Specifically, we examined the molecular mechanisms by which tumor necrosis factor (TNF) influences cancer metastasis. It comprehensively reviews the involvement of fibroblasts, ECM remodeling, immune cells, and soluble factors in creating a pro-metastatic environment. In addition, this review analyzes the therapeutic implications of targeting the TME and highlights the potential strategies for disrupting metastasis and improving treatment outcomes. By advancing the scientific community’s understanding of these mechanisms, this review aimed to facilitate the development of innovative therapeutic approaches to combat cancer metastasis.

## 2 The architecture of the tumor microenvironment

The TME is critical for cancer progression and metastasis. It consists of a complex mixture of cells and structures surrounding the tumor, each of which plays a critical role in cancer development. This section discusses the various elements of the TME and highlights their functions and interactions.

### 2.1 Cellular and non-cellular components

At the heart of the TME, there are various cellular components including CAFs, immune cells, endothelial cells, and pericytes. These are surrounded by non-cellular elements, such as the ECM, signaling molecules, and soluble factors. Among these, CAFs play a significant role in restructuring the ECM, which aids tumor cell movement and invasion (Ten et al., 2024; Xue et al., 2024).

### 2.2 ECM and tumor cells interaction

The ECM provides more than just structural support; it actively communicates with tumor cells. Alterations in ECM composition and stiffness can change cancer cell behavior, affecting their ability to move and invade new tissues. Tumor cells contribute to this process by releasing enzymes known as MMPs. These enzymes break down ECM proteins, clearing a pathway for cancer spread (Hu et al., 2022; Deng et al., 2022).

### 2.3 Immune cells within the TME

Although immune cells typically protect against diseases within the TME, they can be manipulated to assist tumor growth. Tumor-associated macrophages (TAMs) help tumors by dampening immune responses and enhancing the formation of new blood vessels, a process known as angiogenesis (Lu et al., 2022). Additionally, tumor cells can escape immune attack by engaging immune checkpoints to turn off the immune response (Wang et al., 2022).

### 2.4 Angiogenesis in the TME

Tumor growth and metastasis rely heavily on angiogenesis. This process, driven by signals such as VEGF from tumor cells, involves the formation of new blood vessels that supply the tumor with essential nutrients and oxygen. Switching to an angiogenic state is a critical step in the progression of a benign mass to a malignant tumor (Jiang et al., 2020).

### 2.5 Metabolic changes in cancer cells

To thrive, especially in the oxygen-poor environment of the TME, cancer cells often switch metabolic pathways. Metabolic reprogramming term in cancer cells refers to the alterations in cellular metabolism that enable cancer cells to meet the increased energy demands, biosynthetic needs, and redox balance required for rapid proliferation and survival. This includes shifts such as increased glucose uptake and glycolysis, enhanced glutamine metabolism, and fatty acid synthesis, even in the presence of oxygen. These metabolic changes support the anabolic growth of tumors, facilitate adaptation to hypoxic environments, and provide cancer cells with the necessary resources to evade immune responses and sustain metastatic potential (Nong et al., 2020). This allows cells to grow rapidly and outcompete normal cells for resources (Jin et al., 2023). These adaptations create a harsh environment for normal cells, further tipping the balance in favor of tumor growth.

### 2.6 The role of hypoxia

Low oxygen levels or hypoxia in the TME triggers a survival response in cancer cells. HIFs become active under these conditions, turning on genes that help the tumor survive, grow new blood vessels, and spread to other parts of the body. This shows how cancer cells can turn a challenging situation into an advantage (Chen et al., 2022).

### 2.7 Complex communication networks

The TME features sophisticated signaling networks that involve interactions among different cell types. Communication between tumor cells and CAFs can activate pathways such as TGF-β and Platelet-derived growth factor (PDGF), which drive tumor growth and resistance to chemotherapy (Wu et al., 2021). These networks are critical for coordinating the complex behavior of tumors.

In exploring the architecture of the TME, it becomes clear that this environment is not just a passive backdrop for tumor growth, but also an active participant. Interactions within the TME, from cellular communication to metabolic adaptations, create a dynamic setting that fosters cancer progression and spread (Baghban et al., 2020). Understanding these interactions provides valuable insights into the mechanisms underlying cancer metastasis and highlights potential therapeutic targets. By targeting specific components and interactions within the TME, new strategies can be developed to disrupt the supportive environment on which tumors depend, thereby opening new avenues for treatment. The study of the TME is thus not only about understanding cancer better but also about finding new ways to fight it.

## 3 Molecular mechanisms of the TME contributing to cancer metastasis

This section examines the molecular mechanisms by which TME facilitates the spread of cancer from the primary tumor to distant organs. Before diving into specific molecular pathways, it’s important to understand how these mechanisms bridge the basic structural roles of the TME with their direct influence on metastatic progression.

### 3.1 Cell-cell communication and signaling pathways

**FIGURE 2:**
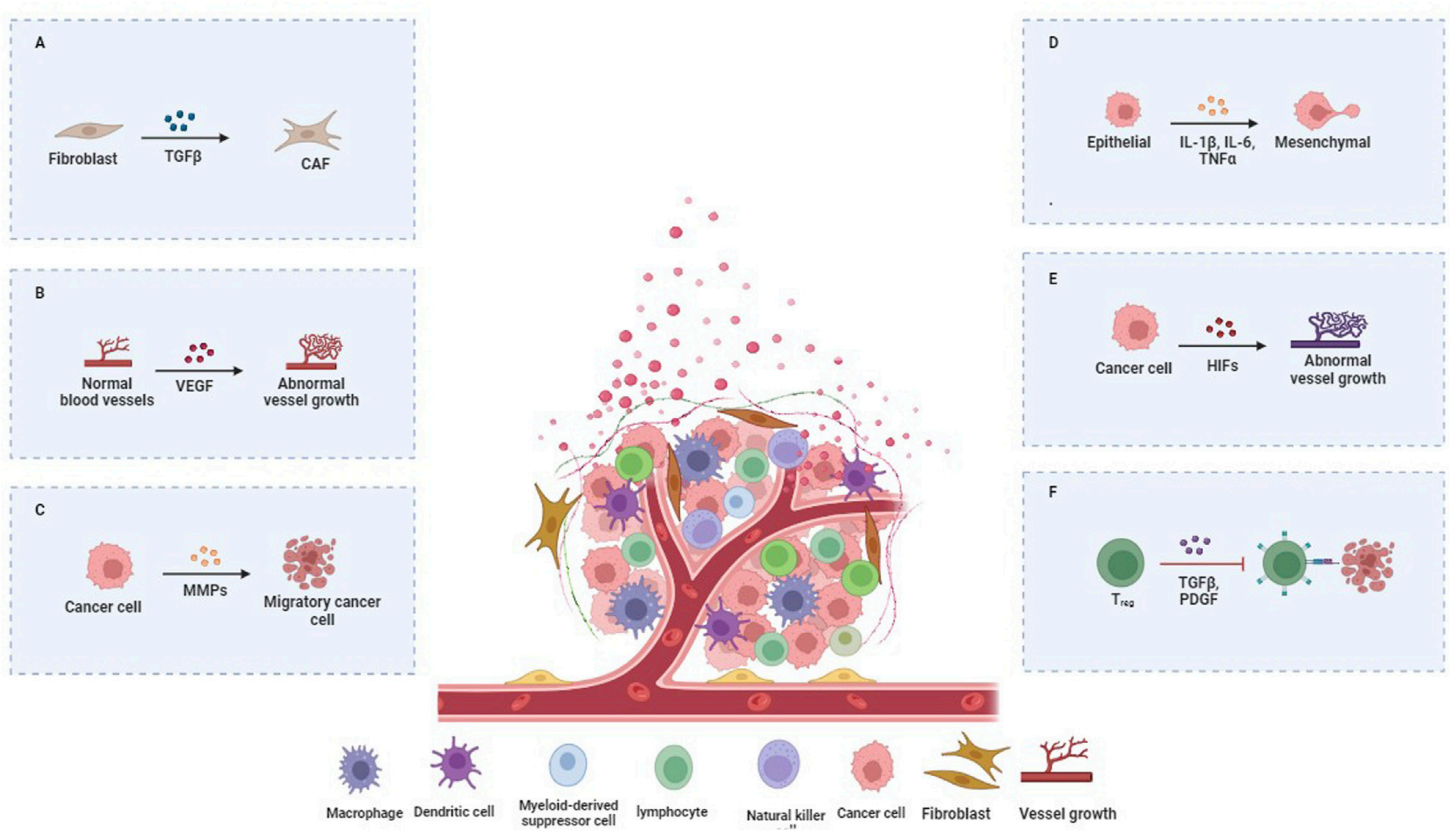
Key Signaling Pathways in TME-Mediated Cancer Metastasis. **(A)** CAFs, immune, and endothelial cells are surrounded by ECM and signals promoting tumor spread. **(B)** Immune cells in the TME can be manipulated to support tumor growth. Tumor-associated macrophages (TAMs) aid tumors by suppressing immune responses and enhancing angiogenesis through signals like VEGF. **(C)** The metalloproteinases (MMPs) enzymes break down ECM proteins, clearing a path for the cancer to spread. **(D)** Tumor cells can escape immune attack by engaging immune checkpoints to turn off immune responses. **(E)** The TME’s signaling networks involve interactions among various cell types. Tumor cells and CAFs activate pathways like TGF-β and PDGF, driving tumor growth and chemotherapy resistance. **(F)** The TME’s signaling networks involve interactions among various cell types. Tumor cells and CAFs activate pathways like TGF-β and PDGF, driving tumor growth and chemotherapy resistance.

The intricate communication between tumor cells and the surrounding stromal cells, including fibroblasts, immune cells, endothelial cells, and adipocytes, represents a pivotal aspect of the influence of the TME on cancer metastasis. This interaction is mediated by cytokines, growth factors, and chemokines secreted by both tumor and stromal cells. Figure 2 illustrates the important signaling pathways involved in cancer metastasis mediated by the TME.

These signaling molecules activate pathways such as TGF-β, Wnt, and Hedgehog, which are pivotal for promoting tumor cell invasion and migration (Neophytou et al., 2021; Jin and Jin, 2020). Table 1 shows the significant signaling pathways involved in TME-mediated cancer metastasis. For instance, TGF-β acts as a double-edged sword, suppressing early tumor development but paradoxically enhancing metastatic potential in advanced stages by inducing epithelial-to-mesenchymal transition (EMT). EMT endows epithelial cells with mesenchymal properties, including enhanced migratory capacity, invasiveness, and resistance to apoptosis, thereby facilitating metastasis (Hao et al., 2019) (Figure 2).

**TABLE 1:** Signaling pathways involved in TME-Mediated cancer metastasis.

Cancer	Genomic alterations	Function	References
Breast Cancer	*BRCA1* and *BRCA2* mutations	Increase the risk of both sporadic and familial breast cancer	Heramb et al. (2018)
	*TP53* mutations	Commonly associated with triple-negative breast cancer (TNBC) can influence treatment responses, especially to checkpoint inhibitor treatment	Ungerleider et al. (2018), Kumar et al. (2024)
	*PIK3CA* mutations	Usually related to hormone receptor-positive breast cancer Enables personalized treatment approaches such as alpelisib	Li et al. (2023)
Lung Cancer	*KRAS* mutations	Associated with lung adenocarcinomas and commonly indicate a poor prognosis	Timar and Kashofer (2020), Wang et al. (2021)
	*TP53* mutations	Related to lung squamous cell carcinomas and affect therapy response and prognosis	Timar and Kashofer (2020), Wang et al. (2021)
	*CDKN2A* mutations	Involved in lung squamous cell carcinomas commonly indicate aggressive behavior and resistance to certain therapies	Timar and Kashofer (2020), Wang et al. (2021)
	*EGFR* mutations and *ALK* fusions	Associated with non-small cell lung cancer (NSCLC) represent a potential target for therapeutics	Shimamura et al. (2022)
Colorectal Cancer	numerous mutations in the *Wnt* signaling pathway components (*e.g., APC*, *CTNNB1*, and *AXIN2*)	Play a crucial role in tumor initiation and progression affect prognosis and therapy response guide targeted therapy decisions	Zhao et al. (2022a)
	microsatellite instability (MSI)	Cause DNA repair mechanism impairment and a hypermutated genome indicating a higher susceptibility to immunotherapy	Zheng et al. (2020)
Melanoma	*BRAF V600E* mutation	A worthy candidate for BRAF and MEK inhibitors	Guadarrama-Orozco et al. (2016)
	*NRAS* and *NF1* mutations	Associated with cutaneous melanomas	Haugh and Daud (2024)
Pancreatic Cancer	*KRAS*, *CDKN2A*, TP53, and *SMAD4* mutations	indicate pancreatic ductal adenocarcinoma (PDAC) affect targeted therapy options	Voutsadakis and Digklia (2023)
	*BRCA1* mutations	A promising candidate for DNA damage response pathway inhibitors	Lai et al. (2022)
Leukemia	*FLT3*, *NPM1*, and *CEBPA* mutations	Significant prognostic parameters for acute myeloid leukemia (AML)	Bystrom and Levis (2023), Falini et al. (2020), Taube et al. (2022)
	*IGHV* and *TP53* mutations	Crucial determinants for the most successful treatment regimen in chronic lymphocytic leukemia (CLL)	Delgado et al. (2014)
Glioblastoma	*IDH1* and IDH2 mutations	crucial for the classification of gliomas	Haider et al. (2023)
	TERT promoter mutations, *EGFR* amplifications, loss-of-function mutations of *PTEN* and *TP53*	Contribute to tumor growth and therapy resistance crucial for establishing more effective and personalized treatment methods	Powter et al. (2021), Li et al. (2023), Wajapeyee and Gupta (2021)

### 3.2 Matrix remodeling and invasion

Another crucial aspect is ECM remodeling by MMPs, which are upregulated in the TME. MMPs degrade ECM components, creating a path for tumor cells to invade neighboring tissues and disseminate through the circulatory system. Furthermore, the altered ECM serves as a reservoir for growth factors, which further stimulate tumor cell growth and survival (Yuan et al., 2023; Chen et al., 2023).

The *in vivo* mechanical properties of the extracellular matrix (ECM) are significant. A stiffer matrix, commonly found in tumors, correlates with enhanced rates of cell division and migration. This is facilitated by the involvement of mechanotransduction pathways, specifically Focal Adhesion Kinase (FAK) and Src family kinases. These pathways activate downstream effectors that stimulate cell motility (Mai et al., 2024).

### 3.3 Immune evasion and modification

The presence of immune cells within the TME can also be a source of immune evasion and modification. These cells, such as TAMs and myeloid-derived suppressor cells (MDSCs), can undergo modifications that support tumor growth and metastasis (Rajbhandary et al., 2023). For instance, tumor-derived factors polarize TAMs into an M2-like phenotype, which supports tumor growth and suppresses antitumor immune response. MDSCs impede the cytotoxic activity of T cells and natural killer cells, thereby reducing immune surveillance and facilitating tumor escape (Li et al., 2022a).

### 3.4 Angiogenesis and vascular modulation

TME also promotes angiogenesis and the formation of new blood vessels, which are essential for tumor growth and serve as conduits for metastasis. VEGF is a key player in this process and is secreted by both tumor and stromal cells (Kim et al., 2022). Elevated levels of VEGF enhance vascular permeability and promote the formation of disorganized and leaky tumor vasculature, which facilitates tumor cell entry into the bloodstream (Janes et al., 2024).

### 3.5 Metabolic reprogramming

Recent studies have highlighted the role of metabolic reprogramming in the TME. Cancer cells often exhibit altered metabolism, characterized by increased glucose uptake and lactate production, even in the presence of oxygen (Warburg effect) (Barba et al., 2024). This metabolic shift not only fuels tumor growth but also creates an acidic TME that impairs immune cell function and promotes tissue invasion (Barba et al., 2024). In the TME, T-cells undergo significant changes that reduce their ability to fight cancer. In the TME, T cells undergo metabolic reprogramming that significantly impacts their functionality. Naïve T cells primarily rely on oxidative phosphorylation (OXPHOS) for ATP production at rest, but upon activation, they switch to glycolysis to meet increased energy demands. Tumor cells compete for critical nutrients like glucose, depriving T cells of essential resources. Additionally, tumor-secreted metabolites, such as lactic acid and prostaglandin E2, disrupt T cell metabolism, creating an acidic environment that inhibits T cell activation. This interplay between tumor and immune cells fosters an immunosuppressive microenvironment, ultimately weakening the antitumor immune response and promoting tumor survival (Nong et al., 2020). In the TME, tumor-associated macrophages (TAMs) experience metabolic reprogramming that affects their function and polarization. Exposure to tumor-derived signals often shifts TAMs from a pro-inflammatory M1 phenotype, reliant on oxidative phosphorylation, to an immunosuppressive M2 phenotype characterized by increased glycolysis and fatty acid synthesis. Hypoxia in the TME stabilizes hypoxia-inducible factor 1-alpha (HIF-1α), further promoting glycolytic activity and the production of immunosuppressive metabolites like arginase-1 and IL-10. Additionally, nutrient competition within the TME alters macrophage metabolism, reinforcing an immunosuppressive environment that supports tumor growth, angiogenesis, and immune evasion (Li and Tian, 2023). The molecular mechanisms through which the TME contributes to cancer metastasis are complex and multifaceted, involving alterations in cell signaling, ECM remodeling, immune modulation, angiogenesis, and metabolic reprogramming (Neophytou et al., 2021). Understanding these interactions provides critical insights into potential therapeutic targets for the prevention and treatment of metastatic cancer.

## 4 The interplay between tumor cells and the TME

The TME is not merely a passive backdrop for the drama of oncogenesis; rather, it is a dynamic participant in the narrative of cancer development and progression (de Visser and Joyce, 2023). Having explored the molecular basis of metastasis in the previous section, we now move to the broader interactive dynamics between tumor cells and their microenvironment. These interactions, as we will discuss, form a critical feedback loop, furthering cancer’s invasive properties.

The TME is not merely a passive backdrop for the drama of oncogenesis; rather, it is a dynamic participant in the narrative of cancer development and progression. Having explored the molecular basis of metastasis in the previous section, we now move to the broader interactive dynamics between tumor cells and their microenvironment. These interactions, as we will discuss, form a critical feedback loop, furthering cancer’s invasive properties.

### 4.1 Bidirectional communication

The interaction between tumor cells and TME is fundamentally shaped by bidirectional communication. Tumor cells secrete a variety of factors, including cytokines, chemokines, and growth factors, which actively recruit and modify the behavior of surrounding stromal cells, including fibroblasts, immune cells, and endothelial cells. Conversely, stromal cells secrete signals that influence the behavior of tumor cells, enhancing their proliferation, survival, and metastatic capabilities (Baghban et al., 2020; Imodoye et al., 2024).

For instance, CAFs, a prevalent component of stromal cells in numerous cancers, secrete growth factors such as fibroblast growth factor (FGF) and hepatocyte growth factor (HGF), which are known to facilitate tumor growth and resistance to therapies (Wu et al., 2022). Additionally, they remodel the ECM, rendering it more conducive to tumor invasion and dissemination (Yuan et al., 2023).

### 4.2 Immune cell modulation

The TME is also characterized by intricate interactions with a multitude of immune cells. Normally, immune cells would detect and destroy aberrant tumor cells. However, in the TME, tumor cells can evade immune surveillance through several mechanisms. One common strategy is the secretion of immunosuppressive molecules, such as PDL1, which binds to PD1 on T cells, effectively “turning off” these would-be attackers (Park et al., 2022; Giraldo et al., 2019).

In addition, tumors frequently manipulate immune cell types, such as macrophages, converting them into tumor-promoting cells that secrete cytokines that support tumor growth and suppress other immune responses. This alteration not only facilitates tumor growth but also contributes to the establishment of a metastatic niche at distant sites (Mirzapour et al., 2023).

### 4.3 Angiogenesis

Angiogenesis, the process of new blood vessel formation, interacts closely with tumor cells and the TME. Tumor cells can secrete angiogenic factors, such as VEGF, which stimulate nearby endothelial cells to form new blood vessels. These vessels provide the necessary nutrients and oxygen to the growing tumors and offer a route for tumor cells to enter the bloodstream, which is a critical step in metastasis (Yang et al., 2024; Fang et al., 2023).

### 4.4 Metabolic interactions

Metabolic interplay within the TME also significantly affects tumor progression. Tumor cells frequently exhibit altered metabolic processes characterized by increased glucose consumption and lactate production. This metabolic shift can result in acidification of the TME, which impairs the function of antitumor immune cells and facilitates their invasive behavior of tumor cells (Shen and Kang, 2018; Benvenuto and Focaccetti, 2024).

### 4.5 Exosome-mediated communication

Recent studies have identified the role of exosomes in communication within the TME. These small vesicles carry proteins, lipids, and nucleic acids. Tumor-derived exosomes can manipulate recipient cells to create a pro-tumorigenic environment (Zhao et al., 2022b). For example, exosomes can transfer oncogenic proteins and miRNAs to other cells within the TME, thereby promoting tumor growth and preparing distant environments for metastasis (Dai et al., 2020).

The complex interplay between tumor cells and the TME exemplifies the multifaceted nature of cancer. This intricate process entails a network of interactions in which tumor cells and the surrounding environment reciprocally enhance their respective detrimental roles in cancer progression. By gaining a deeper understanding of these mechanisms, researchers can identify new therapeutic targets that disrupt these interactions and hinder cancer progression.

## 5 Therapeutic targeting of the tumor microenvironment

TME is a complex network in which cancer cells live and thrive. It includes several types of cells and substances that can either suppress or promote tumor growth. Understanding how these elements interact opens the door to new ways of treating cancer, specifically by targeting the environment in which cancer cells depend (Tiwari et al., 2022). The molecular interactions we discussed earlier provide key insights into why the TME is a viable therapeutic target. With this understanding, we now turn to the practical implications of targeting these components for therapy.

One therapeutic approach involves targeting CAFs. These fibroblasts are “hijacked” by cancer cells to support their growth. They help tumors grow by changing the surrounding structure and encouraging the formation of new blood vessels, which tumors need to obtain nutrients. Researchers are exploring drugs that can stop CAFs from doing this, potentially slowing down, or stopping the growth of cancer (Gao et al., 2024). Fibroblast activation protein (FAP) is a key player in CAFs. FAP inhibitors, such as sibrotuzumab, aim to target FAP expressed by CAFs, reducing their tumor-promoting activity. Another approach involves inhibiting CAF-mediated signaling through the inhibition of pyrroline-5-carboxylate reductase 1 (PYCR1) or hyaluronan and proteoglycan link protein-1 (HAPLN1) using treatments like NADH or hyaluronic acid (Zhang et al., 2024). However, the therapeutic targeting of CAFs remains controversial due to conflicting evidence about their role in cancer progression. Some studies suggest that CAFs can have both tumor-promoting and tumor-suppressing activities, depending on the cancer type or stage, leading to inconsistent therapeutic outcomes. Additionally, the heterogeneity within CAF populations creates challenges in identifying universal therapeutic targets. Gaps in understanding remain regarding the long-term effects of targeting CAFs, particularly concerns about potential adverse effects on normal tissue regeneration and repair (Toledo et al., 2022).

Another strategy focuses on the immune cells within the TME. Normally, the immune system attacks anything that it recognizes as foreign, including cancer cells. However, in the TME, immune cells are often tricked to facilitate tumor growth. New treatments, such as immune checkpoint inhibitors (ICIs), aim to wake up these immune cells so that they can start attacking the cancer again. These treatments have been successful in treating some types of cancer by blocking proteins that help cancer cells hide from the immune system (Chen et al., 2024). For instance, in addition to PD-L1 inhibitors like nivolumab and pembrolizumab, which have been approved for use in several cancers, ongoing studies are investigating candidate molecules such as pidilizumab (Zhang et al., 2024). Despite the promise of ICIs, several controversies and limitations remain in the field. While some patients respond remarkably well to these treatments, a significant portion do not benefit at all, raising questions about why only certain tumors are susceptible. Additionally, ICIs can trigger severe immune-related adverse events, as they can cause the immune system to attack healthy tissues. This limits their widespread use and requires close monitoring of patients. Another challenge is the development of resistance, where tumors that initially respond to ICIs become unresponsive over time. Furthermore, the identification of reliable biomarkers to predict which patients will respond to ICIs remains an ongoing area of research. The lack of understanding of how the broader immune microenvironment influences treatment outcomes also highlights gaps in current knowledge, necessitating further studies to refine these therapies (Meng et al., 2024). Anti-angiogenic therapies targeting blood vessels within the TME are also being used. Tumors need to develop new blood vessels to obtain sufficient nutrients and oxygen to maintain growth. By using drugs that prevent these new blood vessels from forming, such as inhibitors of the VEGF pathway, the supply lines to the tumor can be cut off, which can slow down or stop tumor growth (Liu et al., 2022; Wang et al., 2023). The promising therapeutic role of VEGF inhibitors, particularly bevacizumab, is the subject of ongoing research, as these agents not only inhibit angiogenesis and disrupt tumor vascularization but also impact CAFs, which contribute to the TME (Zhang et al., 2024). However, despite initial successes, anti-angiogenic therapies face significant limitations and controversies. One major issue is that tumors often adapt by activating alternative pro-angiogenic pathways, rendering VEGF inhibitors less effective over time. Additionally, these treatments can lead to hypoxia in the tumor, which promotes a more aggressive tumor phenotype and enhances resistance to therapy. There is also conflicting evidence regarding the overall survival benefits of VEGF inhibitors, as some clinical trials have shown only modest improvements in patient outcomes. Furthermore, the heterogeneity of the TME means that not all tumors are equally dependent on angiogenesis, limiting the effectiveness of these therapies in certain cancer types. Side effects, such as hypertension, bleeding, and impaired wound healing, also present challenges, emphasizing the need for better patient stratification and combination therapies to optimize the use of anti-angiogenic treatments (Wang et al., 2023). Altered metabolism of cancer cells in the TME is another potential target. Cancer cells often change their metabolism to survive under less-than-ideal conditions such as low oxygen levels. Drugs that can interrupt these metabolic pathways are currently being investigated. These could potentially starve cancer cells by reducing their energy supply (Jin et al., 2023). Drugs like metformin, 2-Deoxyglucose, Telaglenastat, Ivosidenib, and Olutasidenib target the altered metabolism of cancer cells within the TME, disrupting key metabolic pathways to inhibit tumor growth (Xiao et al., 2023). Despite the potential of targeting cancer metabolism, several controversies and limitations exist in this field. One challenge is the metabolic flexibility of cancer cells; they can often switch between different energy sources or pathways to evade treatment, reducing the long-term efficacy of these drugs. Additionally, many metabolic inhibitors affect both cancer and normal cells, raising concerns about off-target effects and toxicity. The complexity of the TME adds further difficulties, as cancer metabolism is influenced by various factors, including interactions with immune cells and stromal cells, which can alter treatment responses. Furthermore, while preclinical studies of metabolic inhibitors have shown promise, translating these findings into clinical success has been inconsistent, with limited improvements in patient outcomes in some trials (Xiao et al., 2023). Finally, the use of nanoparticles offers a way to deliver drugs directly to the TME, increasing the effectiveness of the treatment while reducing side effects. These tiny particles can be designed to release their drug load only when they reach the acidic environment of the TME or when they encounter specific enzymes that are more active in cancer tissues (Begines et al., 2020).

Focusing on the TME offers a promising yet complex path for cancer treatment. The intricacies and variations within the TME, not only among different tumors but also among patients with the same type of cancer, present significant challenges. Therefore, personalized therapeutic approaches that incorporate multiple treatment modalities may be crucial for achieving effective outcomes. Ongoing research on the TME and its impact on cancer progression is paving the way for more precise and effective therapies. By targeting the supportive environment on which cancer cells depend, researchers are discovering innovative strategies to fight the disease. This field of cancer research is rapidly advancing and has the potential to enhance future cancer treatment protocols.

## 6 Methodological advances in TME research

Investigation of the TME and its intricate involvement in cancer metastasis has seen a rise in methodological sophistication. Building on our previous discussions of therapeutic targeting, recent innovations in research methodologies have given rise to more precise treatment strategies. These advances also enable a deeper understanding of TME’s role in metastasis, fueling future therapeutic developments (Neophytou et al., 2021). These innovations are led by single-cell RNA sequencing (scRNA-seq), a technique that has transformed our understanding of the variety within the TME. By examining the transcriptome of individual cells, single-cell RNA sequencing (scRNA-seq) has uncovered previously unseen cell diversity and interactions, offering a thorough map of TME complexity. Moreover, scRNA-seq enables the precise identification of rare cell populations and dynamic cellular states that are pivotal in metastasis, which were previously obscured in bulk sequencing analyses. This high-resolution approach has facilitated the discovery of novel therapeutic targets and biomarkers by delineating the cellular heterogeneity and intricate communication networks within metastatic niches (Li et al., 2022b). The application of spatial omics technologies, including multiplexed immunofluorescence and *in situ* sequencing, has further enhanced our understanding of this subject matter. These methods maintain the spatial configuration of the TME, enabling researchers to visualize and interpret the intricate relationships between different cell types and their microenvironment. By preserving tissue architecture, spatial omics provides critical insights into how tumor cells co-opt their surroundings to facilitate invasion and immune evasion. Additionally, the ability to map molecular interactions *in situ* allows for the identification of spatially restricted signaling pathways and cellular niches that are essential for metastatic progression, revealing potential targets for therapeutic intervention. This spatial context is important for understanding how local interactions drive metastatic processes (Lee et al., 2023; Rad et al., 2023).

Live imaging techniques, such as intravital microscopy, have provided a dynamic view of the TME. These techniques permit real-time observation of cellular behaviors and interactions, thereby capturing the essence of metastasis as it occurs. These visual insights have been instrumental in validating and refining our understanding of the molecular mechanisms involved in metastasis. Live imaging has also uncovered transient cellular interactions and migratory behaviors that are critical for the metastatic cascade, which are often missed by static imaging methods. Furthermore, the ability to visualize the dynamic interplay between tumor cells and the immune system *in vivo* offers a powerful tool for assessing the efficacy of immunotherapies and identifying novel strategies to hinder metastatic dissemination (Siminzar et al., 2023).

Computational modeling and systems biology have emerged as valuable tools in TME research (Bartocci and Lio, 2016). By integrating multiomic data, these approaches can simulate complex interactions within the TME, thereby predicting how alterations might affect metastasis. The convergence of computational and experimental methods has opened new avenues for therapeutic discovery.

The integration of organoid and organ-on-chip technologies has facilitated the transition from traditional cell cultures and animal models to a more physiologically relevant platform for studying the TME. These systems replicate the structural and biochemical complexity of tissues, providing a controlled environment for testing drugs and understanding TME-specific responses. These models enable the dissection of intricate signaling networks and cellular cross-talk within the TME, providing a systems-level understanding of the regulatory circuits driving metastasis. Additionally, computational approaches facilitate the identification of key driver nodes and potential vulnerabilities in metastatic pathways, offering a predictive framework for designing targeted therapeutic interventions (Wang and Qin, 2023). Finally, epigenetic profiling demonstrated that the TME can influence the epigenetic landscape of cancer cells, affecting their behavior and metastatic potential. This epigenetic reprogramming has been elucidated using techniques such as ATAC-seq, which enables researchers to explore chromatin accessibility and reveal the epigenetic mechanisms that underlie TME-driven metastasis. Epigenetic profiling has also uncovered the role of tumor-associated stromal cells in shaping the epigenetic landscape, driving the activation of pro-metastatic gene expression programs in cancer cells. Moreover, these insights into TME-induced epigenetic changes highlight new opportunities for therapeutic interventions aimed at reversing the epigenetic modifications that promote metastatic progression (Li et al., 2022c). Regarding the threshold of these methodological advancements, the future of TME research appears to be brighter than ever. The convergence of innovative technologies and enhanced biological insights promises to facilitate the development of novel strategies for combating cancer metastasis, thereby offering the prospect of more effective treatments.

## 7 Clinical implications and translation

The enigmatic relationship between the TME and cancer spread is now increasingly understood thanks to recent clinical insights. As we delve deeper into molecular intricacies, the impact on clinical practices and therapeutic development has become more profound. Acknowledging the TME as a crucial factor in metastasis has revolutionized treatment strategies. Instead of focusing solely on cancer cells, novel approaches have aimed to disrupt the TME. This shift has given rise to therapies that adjust the TME, including immune checkpoint inhibitors and treatments designed to normalize tumor blood vessels.

The field of personalized medicine has identified a new ally in TME research. By profiling the TME of individual tumors, clinicians can develop treatment strategies tailored to the unique microenvironment (Han et al., 2024). This precision approach not only enhances treatment efficacy, but also minimizes adverse effects, thereby advancing the goal of individualized cancer care. The prognostic and predictive values of the TME characteristics cannot be overstated. Biomarkers that reflect the composition and activity of the TME are emerging as powerful tools for clinical decision-making. For example, the density and status of immune cells within the TME can be used to predict patient outcomes and guide the choice of therapy (Siminzar et al., 2023). The fields of surgical and radiation oncology are also influenced by insights into TME (Jiang et al., 2021). An understanding of how these interventions alter the microenvironment and impact metastatic potential is vital for planning treatment and managing cancer patients. The objective is to strike a balance between local control and minimize the risk of systemic dissemination.

Cancer prevention and early detection strategies are beginning to incorporate knowledge of TME. Identification of high-risk microenvironmental features may facilitate early identification of pre-metastatic niches, thereby providing opportunities for intervention before the occurrence of metastasis (Siminzar et al., 2023). In the context of clinical trials, TME has emerged as a key area of focus. In recent years, there has been a growing tendency for clinical trials to be designed in such a way that they assess the impact of treatments on the TME, in addition to more traditional efficacy measures. This shift reflects a growing appreciation of the role of TME in treatment response and resistance (Korkaya and Orsulic, 2020). The translation of TME research into clinical applications holds immense promise in the nexus between basic science and clinical practice. The transition from laboratory to clinical practice is difficult; however, the potential to revolutionize cancer care through a more profound comprehension of the TME is within reach.

## 8 Future directions and perspectives

The TME is a complex system that plays a critical role in cancer treatment. This intricate interplay can lead to the development of targeted therapies and improved patient outcomes. Future research should focus on delineating the cellular and molecular pathways within the TME, identifying, and characterizing the specific roles of various stromal components, and mapping heterogeneity within the TME using single-cell technologies and spatial transcriptomics. Immunotherapy has become a transformative approach in oncology; however, its efficacy varies among patients. Therefore, future research should aim to understand the interactions between cancer cells and the immune system within the TME, which could help develop more effective immunomodulatory therapies. This could involve designing biomarkers that predict response or resistance to immunotherapy, enabling personalized treatment plans. Metabolic reprogramming within the TME is another area of significant potential. Understanding these metabolic shifts could help develop drugs that interrupt energy supply to tumors or inhibit their metabolic flexibility, which is particularly effective in targeting metastatic cancer cells. Furthermore, research on the interactions between microorganisms, cancer cells, and the immune system may reveal new therapeutic targets or ways to enhance existing treatments. Additionally, it is essential to address the ethical, legal, and social implications of TME research on an ongoing basis. Policies and guidelines must evolve in tandem with scientific advancements to ensure the ethical and equitable realization of the benefits of TME research. In conclusion, TME research holds enormous potential as it employs advanced technologies and interdisciplinary methodologies to explain the biological intricacies of the TME.

## 9 Conclusion

Research on the TME has revealed its critical role in cancer development and metastasis. The TME promotes primary tumor growth by facilitating complex molecular interactions and orchestrating the spread of cancer cells to distant sites. The combined activity of key components, including stromal cells, ECM, and signaling molecules, enhances the invasive potential of cancer cells and influences the immune response. Recent breakthroughs have emphasized the potential therapeutic benefits of targeting the TME, suggesting a strategy to hinder the spread of cancer at various stages. As research progresses, a comprehensive understanding of the intricacies of TME is likely to improve these approaches, offering more effective and precise cancer treatments. This review emphasizes the importance of ongoing TME research for discovering novel treatment strategies that may hinder or even reverse the harmful processes of cancer metastasis.
